# Creating Change: Art Activism and Leadership Development

**DOI:** 10.1002/yd.20652

**Published:** 2025-02-24

**Authors:** Jessica A. Cruz, Leighton E. Vermont, Sally R. Watkins

**Affiliations:** ^1^ Christopher Newport University Newport News Virginia USA; ^2^ Educational Leadership and Policy Studies Florida State University Tallahassee Florida USA

## Abstract

This article advocates the inclusion of the arts, and specifically, art activism, as a beneficial pedagogical approach to leadership development. Focus is given to using examples of art activism to introduce the student leader activist identity continuum (SLAIC), the exploration of leadership identity, and situating activism as transformative leadership. This article situates art activism as a means of transformative leadership and advocates using activist art to introduce and support movement along the SLAIC.

## 1 Introduction

The aesthetic disciplines of visual arts, music, dance, theater, and creative writing are woven into our lives and infuse popular culture. Historically, social issues and the arts are constant threads in our experiences. We experience both differently, yet much like popular culture, they are a commonality of life (Beyer 2000). Individuals and groups regularly try to create change through collective action or activism in response to pervasive social issues. The experiences, perspectives, and voices of those acting have been and continue to be captured through the visual arts or arts activism, a manifestation of transformative leadership. In this article, we situate arts activism, a component of popular culture, as leadership, introduce the student leader activist identity continuum (SLAIC; Bruce et al. 2019), and bring attention to activism as a form of transformative leadership so that as students seek to address the social issues of today, leadership educators and practitioners can contribute to developing their activist leader identity and potentially shape their capacity and efficacy as transformative leaders.

### 1.1 Art and Leadership

Often referred to as an art and a science, leadership is not typically associated with the aesthetic disciplines. The arts, artists, and their products are rarely tied to leadership, even though successful leadership is often framed as an art (Caulfield et al. 2021). Guthrie et al. (2021) argued we need to engage collectively to address wicked problems (Rittel and Webber 1973) and suggested that leadership practiced as an art is the approach we should take because such problems cannot be solved by applying known strategies. Leadership as art offers solutions that arise through observation, envisioning, innovation, creativity, adaption, and collective action. People from all walks of life contribute to collective action, but the arts and artists are uniquely situated to highlight social issues and disrupt systems that perpetuate them. When Astin and Astin (2000) wrote *Leadership Reconsidered*, they identified several value ends of leadership, including cultural enrichment and creative expression, which are concepts closely linked to the arts and popular culture.

### 1.2 Transformative Leadership and Art Activism

Burns (1978) advocated transforming leadership as a revolutionary approach to create real change, markedly shifting social system attitudes, norms, institutions, and behaviors. Activism can transform negative and oppressive dynamics of social constructs by building and connecting the community (Johnson 2016). Personal passions and shared purposes motivate and inspire activists to engage in the leadership process. Activists respond to the call to act by organizing, allocating resources, and mobilizing others to address inequities, dismantle oppressive systems, and transform communities (Ganz 2009; Trueba 1999). Transformative leadership centers on social justice, positive change, and participatory action to achieve a truly democratic society by increasing opportunities, embracing diversity, and stimulating civic responsibility (Shields 2020).

The most obvious representation of influence via activism is typically experienced through public and physical acts of activism, such as marches, sit‐ins, boycotts, strikes, and picketing, but this is a limited view of activism. Bradley and Esche (2007) posed the question, “How can artists participate meaningfully in social struggles” (p.10). One answer is *art activism*, an approach they situated as the, “…intersection of art and political activism” (Bradley and Esche 2007, 11). Art has been a part of human life and culture for generations, recording events, portraying opinions, advancing a point, and influencing others toward a certain goal. Much like activism, the creative arts are based on critical inquiry and thinking and present an opportunity for activism to move beyond marching and protest to communicating the message of a movement, generating dialogue, and expressing a collective identity (Campana 2011).

Activists engage both through formalized roles and more informal spheres of influence. For artists, activism can lean heavily in the informal realm, and most often, their work is integral to their ability to influence others to respond to the call to act. Activist artist's efforts range from bringing awareness to a topic by highlighting an issue in their creative endeavor to building communities of practice, such as performance artists focused on creating change (Adler 2006). Through their art, artists introduce ideas and perspectives that perpetuate or disrupt thinking, transforming ways of knowing and doing when seen and interpreted by an audience. Mangione (2020) noted how art impacts us and how we impact art is a cycle that can shape systems. Thus, art can be a form of activism, and if activism seeks to create change, it is a form of transformative leadership; it is reasonable to connect art as a form of leadership, and the impact artists can have as leaders. Adler suggests that the creativity and passion of artists and the willingness to imagine possibilities are needed for 21st‐century leadership and beyond.

Art typically serves many functions for the artists and the observer; as a means of social change, the arts can raise awareness of issues, make a statement about a topic, generate dialogue, influence others’ perceptions of an issue, and bring similarly minded people together for collective action. Art can bring the unimaginable and hidden to the forefront and inspire leadership outcomes, including empowerment, mobilizing others, collaboration, consensus building, and transformative change (Lewis 2020). Artists engage in the creative process by establishing a vision, taking risks, navigating obstacles, and negotiating the complexities of bringing a vision to life. Artists can inspire others, unite people, and shape how people think and view the world. Activist artists use their voices and power to further develop their opinions and social positions while encouraging those around them to follow their path.

Activist artists engage in transformative leadership using their talent and creativity to assert influence. For instance, Pablo Picasso, the famed visual artist, painted *Guernica*, which depicts the devastation of war in Spain and encouraged an end to the needless violence. Met with negative reviews, *Guernica* was considered significant to the point of being presented to the United Nations to end the war. However, when the time came for the United States to discuss involvement in the war, the painting was hidden because of the intended message of the work and the influence it carried (Cohen 2003). This interaction between the world's power collective, the United States, and a representative painting demonstrates the potential impact of creative works for activist artists to contribute to leadership to address social issues.

Artists also step into the activist‐leader role when they recognize that their creative works can elicit reactions such as nostalgia, disgust, shock, and other emotions (Artists at Risk Connection 2023). Activist artists assert their influence by purposefully creating pieces and productions that impact people. This ability to impact others through their art is akin to having influence and potential to shape culture. Activist artists do more than create art for consumption or aesthetic appeal. They embrace their ability to be transformational leaders and shape culture by presenting works with intentional and strong purposes to raise support or opposition to cultural values, traits, and practices and call others to action (Tending Our Roots n.d.).

Furthermore, Bain (2022) asserted artists need to believe in their purpose, be confident in their direction, and set themselves up to be leaders in their field. Working from their personal “why,” an artist who employs their creative talents to spread a message, either in opposition or in support of something, to create change and call others to act, has become an activist. Once an activist artist collaborates with others to address inequities, dismantle oppressive systems, and transform communities, they become a transformational leader.

## 2 Pedagogy

Recognizing and acknowledging art activism as leadership creates opportunities for incorporation into leadership learning that aids the growth of leadership skills to fully harness leadership potential (Northwest Executive Education 2024). Leadership educators can incorporate arts‐based experiences into the curriculum, from sharing examples of art activism to opportunities for students to create activist art themselves, individually or collectively.

### 2.1 Art Activism in Leadership Learning Spaces

Using art activism and activist artists as the topic of a discussion prompt or a case study is a starting point for educators and practitioners. We have already introduced one example, the work of Pablo Picasso, in this article. The following section highlights other ways to infuse art activism into leadership learning spaces.

First, *community‐based street art* is a readily available and accessible example of art activism existing in public spaces; street art can be a form of protest and public activism that draws attention to issues and the communities being affected, with artists often being members of the affected community (Riggle 2010). Locating works in the community removes the traditional boundaries (e.g., galleries, museums, and private collections), to engage with the art and potentially removes barriers such as social class, expanding the artists’ exposure and influence. Specifically, the street art culture of a call‐and‐response piece in which multiple community members interact with a singular piece, building upon one another, allows inclusive collaboration and non‐traditional communication (Johnson 2016). Street art places individual experiences and emotions to be seen by the public when they otherwise might not be noticed or understood. Street art offers multiple ways to approach the conversation of art activism as leadership and draw connections to transformational leadership.

Shifting the focus to activist artists, we highlight Barbara Jones‐Hogu, who created a piece entitled “Unite” as a call to action for the black Americans in her Chicago community. This piece was created within the artist's collective AfriCOBRA, which Jones‐Hogu helped found, to unite like artists to create work within the African American perspective, reflect on past struggles, display current efforts, and work toward an improved future during the racial disparity of the civil rights movement (Serrant 2020). As noted, “Unite” itself can be understood through, “…[their] rows of similarly raised fists and solemn faces, many in profile, form a rhythmic pattern atop a single, undifferentiated black mass—their unified body,” (Smithsonian n.d.). As intended, the visualizations, shapes, words, and colors stir feelings in the viewer. When looking at the observer's reaction and interpretation, the intentions were met, both in the empowerment it provided to the black community and the anger it inspired in the opposing white community, which only encouraged the revolutionary fight and discourse over racial equality. Jones–Hogu and AfriCOBRA are starting points for case study development and discussion.

Furthermore, art can be seen in many forms, as discussed earlier, and appreciated even by those who may not realize their relationship with art and be influential just the same. This can be seen in everyday common encounters such as *books and movies*, both art forms through literature and film. The *Hunger Games* novel and film series is one example of activism and acknowledgment of a social issue (Collins 2014). Simmons (2012) explores and emphasizes the ways in which the *Hunger Game*s series acts as a call to action for its audience due to the particularly jarring political representation Collins intentionally frames the story as, resensitizing society to the issues at play in our current world. By placing the realistic issues in a fictional space, the audience can digest and understand the information more easily and then draw the connections between fiction and reality, inciting social responsibility and community activism. This then places authors, screenwriters, filmmakers, and actors as leaders influencing the masses, especially in a series like *The Hunger Games*.

Music is another form of art activism in everyday life, specifically Protest Music. One example is the jazz song “Strange Fruit,” performed by Billie Holiday (Sadrazam and Bayraktar 2023). This song was influential during the civil rights movement, acting as a means for determining political unrest by both acknowledging the issues, capturing the emotions of this difficult time, and providing a space to appreciate the cultures and community of African Americans. The dark and moody lyrics and melody, paired with the eight‐piece African American orchestra, allowed Holiday to build a cohesive and influential piece that expressed her own emotions about being a black woman in 1930s America. The way Holiday presented the emotions and social issues of the time allowed her work to be appreciated, studied, and iconized throughout history.

### 2.2 Art Activism as an Approach to Activist Identity Exploration

Leadership educators understand how successful collective efforts to affect change or activism rely heavily on individuals’ leadership identity, capacity, and efficacy within groups (Guthrie et al. 2021). Like leadership identity development, student leader activist identity development occurs on a continuum with individuals moving across the identities as the issue, context, and community change.

Bruce et al. (2019) captured this evolution of identities from learner to ally, advocate, and activist in the Student Leaders Activist Identity Continuum (SLAIC). As students move along the SLAIC continuum, they become transformative leaders who interrogate systems and structures from an individual and public good perspective while centering justice and equity (McKee and Bruce 2020). Incorporating art activism into leadership development is one way to situate and move individuals, artists, or art observers along the SLAIC to cultivate transformative leadership (Bruce et al. 2019).

An example of an activist artist who demonstrates movement across the continuum is Angela Davis Johnson (2016), who seeks to give a voice to underrepresented populations in her artistic work. Her art focuses on the “…missing and invisible—Black women who do not make the news headlines” (p. 28). She uses her talent to “…make the invisible seen” (p. 28). Johnson's article, *The Artist‐Activist: History and Healing Through Art*, beautifully demonstrates her journey across the SLAIC from learner to activist.

Leadership educators and practitioners should encourage students to reflect on their leadership identities and develop their personal reflective practices as a component of leadership development (Burns et al. 2024). The SLAIC offers a framework for locating the individual as a learner to ally, advocate, and activist identities. After an introduction to the SLAIC (Bruce et al. 2019) and engaging with the Johnson (2016) article, critical reflection is an excellent way for students to position themselves on the continuum; students should distinguish the aspects of each identity that they recognize in themselves. Going a step further, the reflective practice offers an opportunity to explore the SLAIC identities they would like to develop. Completing the reflection‐before‐action aspect of Edwards’ (2017) four‐dimensional reflective process that expands on Schön's (1983) two‐dimensional reflection‐in‐action and reflection‐on‐action process supports students situating themselves on the SLAIC continuum and future planning for movement on the continuum.

### 2.3 Making Space for Activist Art

Beyond observing art and reading about artists and then reflecting on personal identity, envisioning and creating activist art offers the opportunity for students to become activist artists and transformative leaders. For a deeper learning opportunity, working individually or in groups, students can establish their “why” or purpose, then explore the topics connected to their purpose (learner), seek out opportunities to share space with communities shaped by the topics, including how the arts are present or missing in those spaces (ally), move to influence circumstances that shape the experience of people in those communities by highlighting existing arts activism or encouraging the incorporation of arts activism (advocate), and seek to change policies and structures that negatively impact communities and/or limit the arts as a legitimate means of activism (activist). A natural next step would be producing activist art or with communities to produce arts‐based activism. The outcomes of incorporating art activism into leadership courses are numerous and tied to how the instructor presents the topic and the assignments related to art activism. Educators can implement projects where students can create their own or study an art activist piece. Students might give a detailed analysis of how the leadership message is communicated and the theories and concepts present. By reviewing the analysis, educators can assess the application of leadership learning.

## 3 Recommendations and Implications

These are some starting points for a leadership educator and practitioners interested in bringing art activism into their leadership development portfolio. Working with libraries, museums, and the internet, giving students access to activist art is not an obstacle in most situations. Better yet, many examples of activist art may be found in your local communities or on your campuses in various departments.

Students who identify as artists might readily embrace the opportunity to engage in art activism and move on to the SLAIC as they negotiate the subject matter, explore different mediums and techniques, target audiences, and envision the completed creative product. Of course, not everyone identifies as an artist or activist, so introducing art activism in the leadership development space offers opportunities to explore social issues, develop observational skills, inform personal perspectives, and engage in discourse, all potentially contributing to movement on the SLAIC. Educators and practitioners should understand that not all individuals will identify as artists or activists; thus, facilitators must provide support and adapt as some might feel uncomfortable exploring the different identities and highlighted issues.

Similarly, not all leadership development practitioners will feel confident incorporating art activism as a leadership learning tool. This is a natural aspect of adding art activism to leadership development pedagogy when your area of expertise is leadership, not the creative arts. Educators may feel they are not well‐versed in interpreting the artists' message. But remember, artists often speak to the piece or their purpose via an artist's statement which can offer insights and prompt an interpretation. Additionally, what is taken from a creative work is connected to the participant's experiences. Leadership educators and students can legitimately make sense of creative work in how they see or experience it through the lens of leadership learning and explore the meanings together.

Finally, consider this an opportunity to partner with arts community members. Collaborate with creative artists, activist artists, and arts educators to inform your approach and/or invite them to join you as a co‐creator of the experience. Their creativity and insights into historical aspects of art activism, artists’ methods, and techniques will only enhance the experience for everyone involved.

## 4 Conclusion

Art activism is leadership. Historically, the creative arts have played a role in how communities both influence and are influenced by society. Art activism has been undervalued as an effective means of drawing attention to an issue or topic, facilitating change, and transforming communities. Shining a spotlight on activist artists and their work while introducing the Student Leader Activist Identity Continuum is a creative way to explore leader identities, activism, and transformational leadership. With the acknowledgment that activist artists can be transformative leaders, avenues are opened for emerging leaders to realize their potential and for followers to be given a unique opportunity to explore alternative perspectives. Leadership educators must center arts‐based leading formats and encourage creative artists to explore their leadership identity, capacity, and efficacy.
